# Systemic inflammation and emotional responses during the COVID-19 pandemic

**DOI:** 10.1038/s41398-021-01753-5

**Published:** 2021-12-09

**Authors:** Odessa S. Hamilton, Dorina Cadar, Andrew Steptoe

**Affiliations:** Behavioural Science and Health, University College London, London, UK

**Keywords:** Predictive markers, Depression

## Abstract

The impact of the COVID-19 pandemic on population mental health is of global concern. Inflammatory processes are thought to contribute to mental ill-health, but their role in experiences of psychological distress during the pandemic has not been investigated. We tested the hypothesis that elevated inflammatory biomarkers (high-sensitivity plasma C-reactive protein [CRP] and plasma fibrinogen) measured pre-pandemic would be positively predictive of increased depressive symptoms experienced during the pandemic. Data were analysed from the English Longitudinal Study of Ageing (ELSA), with 3574 individuals aged >50 for CRP and 3314 for fibrinogen measured in waves 8 (2016/17) or 9 (2018/19). Depressive symptoms were measured with a short version of the Centre for Epidemiological Studies Depression Scale (CES-D) pre-pandemic (2016–2019) and during the pandemic (June/July 2020). Participants with higher baseline CRP concentrations had 40% higher odds of developing depressive symptoms during the pandemic (OR_adjusted_ = 1.40, 95% CI 1.12–1.73, *p* = 0.003) after full adjustment. Fibrinogen concentrations were also associated with depressive symptoms during the pandemic (OR_adjusted_ = 1.23, 95% CI 1.04–1.46, *p* = 0.019), but this association was no longer significant after controlling for lifestyle factors (smoking status, alcohol consumption and physical activity). In this large population study, systemic inflammation measured 1–3 years pre-pandemic was associated with greater depressed mood during the early months of the pandemic. This finding is consistent with the hypothesis that higher levels of inflammation increase the vulnerability of older people to impaired mental health in the presence of the widespread stress of the COVID-19 pandemic.

## Introduction

﻿The outbreak of the severe acute respiratory syndrome coronavirus 2 (Sars-CoV-2) has led to over 7,600,000 infections of the coronavirus disease (COVID-19) within the UK to date, with 231,550,000 cases worldwide, and a mortality rate among the infected exceeding 2% [1]. The mental health sequelae of the pandemic have become a distinct public health concern [2, 3]. Older adults are among those most vulnerable to fatal incidence of COVID-19 [4], which has led to intense fears of contagion and a heightened awareness of individual fragility. Reports of affective responses have been diverse, from emotional distress, depression, irritability and insomnia to fear, anxiety, despair, guilt and anger [5]. The population worldwide has been subjected to intrusive pandemic containment measures intended to limit pathogen transmission, reduce prognostic severity and minimize mortality. Containment measures have limited daily routines, such that social and economic activity have been substantially reduced, whereas access to healthcare and care provisions have been interrupted [6]. These mitigation efforts have come at the expense of psychological wellbeing [7], with a rise in psychosocial stressors ranging from social isolation and financial insecurity [8] to increased rates of domestic discord [9]. Equally, harmful behaviours such as high-risk alcohol consumption [10], dysfunctional eating [11] and medical care avoidance [6] have been on the rise. The proliferation of pandemic-related stress has raised concerns over the psychological vulnerability of older individuals [12].

COVID-19 has resulted in a dislocation of people’s lives that has had very broad effects. Studies on the emotional responses to earlier epidemics have offered insight into the deleterious impact of highly virulent infectious disease on community mental health that impacts sectors of the population differently [13, 14]. Further, given pre-pandemic inequalities in mental wellbeing, similar disproportionate patterns of vulnerability were anticipated during COVID-19 [15]. Research on responses to the COVID-19 pandemic has exposed disparities in the distribution of distress, the severity of mental illness and variation in the magnitude of change from pre-pandemic status [16]. Demographic factors contributing to effects were found to explain this variation, with lower social status groups, females, ethnic minorities, the disabled and those with pre-existing physical or mental conditions being at greatest risk to adverse emotional responses [4, 5, 15, 17].

Heightened inflammation is a hallmark of advancing age [18] with ‘inflammaging’ recognized as a phenomenon linked with numerous health problems [19]. Systemic inflammation may also contribute to psychological distress and risk of depression, with the dysregulation of pro-inflammatory cytokines predicting psychological distress [20, 21] and future psychopathology [22]. Multiple markers of inflammation including C-reactive protein (CRP) and fibrinogen, have been found to predict poor course of depression in some studies [23, 24], and both have been established as sensitive markers of inflammation in relation to factors, such as socioeconomic status, social isolation and loneliness [25–27]. However, meta-analytic surveys of existing evidence have concluded that links between CRP concentration and future depression in both adults and children are weak and inconsistent [28, 29]. Such associations may be more likely to emerge under conditions of severe stress. It is plausible, therefore, that heightened antecedent inflammation primes vulnerable individuals to increased depressive symptoms in the face of pandemic-related challenges, particularly when those psychosocial stressors are perceived as unpredictable and uncontrollable. The pandemic may function as a catalyst for priming neuroimmune dysregulation to increase risk of depressive symptoms. However, our understanding of these processes is limited by a lack of studies linking pre-pandemic inflammation with mental health outcomes during the COVID-19 pandemic. The current study, therefore, aimed to examine the incidence of depressive symptoms in English older adults during the COVID-19 pandemic, while taking into consideration levels of inflammatory markers and depressive symptoms before the pandemic. We postulated that individuals with higher systemic inflammation pre-pandemic would present with higher depressive symptoms during the pandemic.

## Methods and materials

### Study design

**Fig. 1:**
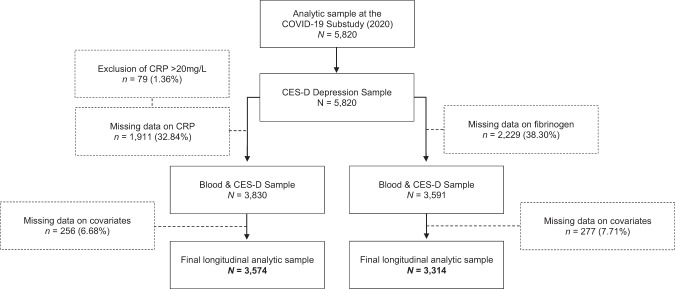
Flow chart of the COVID-19 Substudy analytic sample. Data source: ELSA waves 8/9 (2016/19) and COVID-19 Substudy (2020).

Fully anonymized data were drawn from the English Longitudinal Study of Ageing (ELSA), a nationally representative, multidisciplinary prospective observational study of the English population aged 50 years and older [30]. The COVID-19 Substudy started in June 2020 to capture a robust selection of psychosocial experiences during the pandemic, using an online platform or computer-assisted telephone interviews. The present study used data from the COVID-19 Substudy (2020) and ELSA wave 8 (2016/17) and wave 9 (2018/19). The COVID-19 Substudy had a 75% response rate (*n* = 7040), of which 5820 were core respondents, whereas the remaining were non-core (younger partners of the core respondents from whom data were collected). There were no missing data on depressive symptoms. Of the 5820 core respondents, 3830 had measures of CRP and 3591 of plasma fibrinogen in waves 8 and 9. After exclusions on missing data for covariates, the final sample was 3574 for CRP analyses and 3314 for fibrinogen (see the formation of each analytic sample in Fig. 1).

### Depressive symptoms

The eight-item Centre for Epidemiologic Studies Depression Scale (CES-D) [31] was used to assess depressive symptoms ‘over the past week’ in the COVID-19 Substudy and ELSA waves 8 and 9. However, one item (i.e., “felt sad much of the time…”) was unintentionally omitted from the COVID-19 Substudy, so for comparability, an analogous seven-item scale was calculated for the pre-pandemic waves 8 and 9. All items were scored on a binary scale (1 = ‘yes’; 0 = ‘no’) and positively worded items were reversed scored. Scores were summed to generate a total score of depressive symptoms ranging from 0 to 7, with a higher overall score indicating greater depressive symptoms. The internal consistency (Cronbach’s α) in this sample was 0.75 across waves 8 and 9, and 0.80 in the COVID-19 Substudy, indicating good scale reliability. A threshold of ≥4 was used to determine depressive symptoms caseness, which produces comparative results to the 16-symptom cutoff in the 20-item CES-D scale [32].

### Inflammatory biomarkers

Blood samples were collected by study nurses in participants’ homes and were analysed for two inflammatory markers: high-sensitivity plasma CRP (mg/L) and plasma fibrinogen (g/L). Samples were collected for half of the participants in wave 8 and the remaining during wave 9; data were then combined. Blood samples were dispatched to the Royal Victoria Infirmary (Newcastle-upon-Tyne, UK) for processing and analysis. Samples received more than 5 days after collection were discarded. Blood sampling exclusion criteria included coagulation, haematological disorders, being on anticoagulant medication or having a history of convulsions.

High-sensitivity plasma CRP (mg/L) was assayed using the N Latex CRP mono Immunoassay on the Behring Nephelometer II analyser (Dade Behring, Milton Keynes, UK). Intra- and inter-assay coefficients of variation were <2%. The lower detection limit of the assay was 0.2 mg/L. CRP values >20 mg/L were excluded from analyses (*n* = 79), as these were taken to reflect acute inflammatory processes rather than chronic inflammation. Using a well-recognized clinical demarcation of inflammation in the adult population [33], CRP was dichotomized low (<3 mg/L) and high (≥3 mg/L).

Plasma fibrinogen (g/L) was analysed using a modification of the Clauss thrombin clotting method on the Organon Teknika MDA 180 coagulation analyser (Organon Teknika, Durham, USA). The intra and inter-assay coefficients of variation were <7%. The lower detection limit of the assay was 0.5 g/L. Fibrinogen had a normal distribution and was treated as a continuous variable, with higher values indicating greater levels of inflammation.

### Covariates

Variables considered likely to confound the analyses were selected a priori, comprising: *sociodemographic variables*: age, sex, education (recoded from seven items into four categories: 1 = ‘Higher Education’; 2 = ‘Tertiary Education’; 3 = ‘Secondary/Primary Education’; 4 = ‘Alternative/No Education’) and wealth (indexed by quintiles of total household wealth; i.e., financial wealth, property value [minus mortgage], business assets and physical wealth, net of debt); smoking status, alcohol consumption (‘less than daily’ vs. ‘daily [5–7 per week]’) and physical activity (a five-point classification indicating the amount of moderate or vigorous physical activity); *clinical variables*: plasma triglycerides, high-density lipoprotein (HDL), low-density lipoprotein (LDL) and limiting longstanding illness (a dichotomous variable indicating the presence of any long-term illness, disability or infirmity that limits activity).

### Statistical analyses

Descriptive statistics are reported as means (M) and standard deviations (SD) or *n* (%). Longitudinal associations between inflammatory markers at baseline (combined waves 8 and 9; 2016/19) and depressive symptoms during the COVID-19 Substudy (2020) were assessed with logistic regressions. Separate analyses were carried out for each inflammatory marker. We computed odds ratios (OR) with 95% confidence intervals (CI) for the presence of elevated depressive symptoms among people with high CRP, with the reference category being low CRP. The analyses of fibrinogen report the OR of elevated depressive symptoms per unit increase in fibrinogen concentration. The basic model (model 1) adjusted for pre-pandemic depressive symptoms only. Subsequent models additionally adjusted for age and sex (model 2), education and wealth (model 3), smoking status, alcohol consumption and physical activity (model 4), and triglycerides, HDL, LDL and limiting longstanding illness (model 5). The final model (6) included all covariates. Data management and analyses were conducted in Stata MP 14.1, TX USA.

### Sensitivity analyses

The first sensitivity analysis tested whether the associations found in the main analyses depended on the binary classification of elevated depressive symptoms (i.e., using the CES-D threshold); instead, we analysed the continuous CES-D scores. The results are presented as standardized regression coefficients with standard errors (SE). We suspected that exposure to COVID-19 may cause an overestimation of emotional responses, so the second sensitivity analysis tested whether the results remained unchanged when participants with possible COVID-19 infection were excluded. We assessed exposure to COVID-19 in two ways. First, participants were asked whether they had been hospitalized for COVID-19. Second, we evaluated the presence of at least two of the three core coronavirus symptoms defined by the UK National Health Service (NHS): ‘high temperature’; ‘new continuous cough’; and ‘loss of sense of smell or taste’. Those hospitalized or those meeting the NHS criteria for core symptoms were categorized as possible COVID-19 cases. In the third sensitivity analysis, we reassessed the exclusion of very high CRP values on the basis of arguments put forward by Giollabhui et al. [34]. The regression models were therefore repeated, including individuals with CRP values ≥20 mg/L in the elevated depressive symptom group. The fourth sensitivity analysis evaluated whether associations between CRP and later depression depended on the binary division of CRP into normal and high categories. We therefore included continuously distributed CRP values into the regression models. Logarithmic transformation was performed on the positively skewed CRP scores to normalize the distribution. Fifth, we added body mass index (BMI) as an additional covariate. BMI was not included in the primary analyses, because conditioning on BMI may introduce collider stratification bias [35, 36] and because the sample size was reduced through missing data on height and weight. The sixth sensitivity analysis repeated the analyses with alcohol intake modelled across the full range of consumptions (eight points from ‘almost daily’ to ‘not at all in the past 12 months’) instead of categorization as a binary variable. Seventh, we considered the possibility that the individual’s exposure to different types of stress during the pandemic was responsible for the results. We therefore identified a suite of measures of personal exposure to the coronavirus (a combined measure of NHS core symptoms, personal hospitalization, or household member hospitalization and/or death due to coronavirus), together with the financial impact of the pandemic (current personal financial circumstance on a five-point scale [‘much worse off’ to ‘much better off’, as compared to pre-pandemic status) and a difficulty in accessing services during the pandemic (including access to a bank/cashpoint, supermarket, hospital and/or pharmacy on a four-point scale [‘easy’; ‘difficult’; ‘unable’; ‘unwilling’]). These variables were added as covariates to test whether the association between pre-pandemic inflammation and depressive symptoms during the pandemic was reduced when these factors were taken into account.

## Results

Participant baseline characteristics are displayed in Table 1. CRP and fibrinogen were positively correlated (*r* = 0.481, *p* < 0.001). There were no notable differences in participant characteristics between the CRP (*n* = 3574) and fibrinogen (*n* = 3314) samples. Participants were ~43% male and ~57% female, with a mean age of ~69.89 (±8.40; range = 52–90). In both samples, the majority of participants were non-smokers (81.59%/81.50%) and only around one in five drank alcohol on most days of the week. Two-thirds reported no longstanding limiting illness (70.90%/70.28%), whereas over a third were engaged in moderate or vigorous physical activity (42.97%/42.58%). A small number of participants had been exposed to the coronavirus, 82 were symptomatic (2.29%/2.48%) and 12 had been hospitalized (0.34%/0.36%). Before the pandemic, 7.19% (CRP analysis) and 7.45% (fibrinogen analysis) had depressive symptoms above threshold, and this increased to 16.03%/16.32% during the pandemic, confirming a large increase in incidence of significant depressive symptoms.

**Table 1:** Sample characteristics for the CRP and fibrinogen analyses.

Variable		CRP (*n* = 3574)			Fibrinogen (*n* = 3314)		
		Obs.	Mean (SD)/*N* (%)	Range	Obs.	Mean (SD)/*N* (%)	Range
Age		3574	67.91 (8.38)	52–90	3314	67.87 (8.42)	52–90
Sex	Male	1548	43.31%		1439	43.42%	
	Female	2026	56.69%		1875	56.58%	
Education	Higher education	1492	41.75%		1362	41.10%	
	Tertiary education	413	11.56%		389	11.74%	
	Secondary/primary education	910	25.46%		850	25.65%	
	Alternative or no education	759	21.24%		713	21.51%	
Wealth	Lowest quintile (1)	428	11.98%		411	12.40%	
	2nd Quintile	558	15.61%		514	15.51%	
	3rd Quintile	805	22.52%		747	22.54%	
	4th Quintile	917	25.66%		848	25.59%	
	Highest quintile (5)	866	24.23%		794	23.96%	
Smoking status	Non-smoker	2916	81.59%		2701	81.50%	
	Smoker	658	18.41%		613	18.50%	
Alcohol consumption	Less than daily	2886	80.75%		2680	80.87%	
	Daily (5–7 per week)	688	19.25%		634	19.13%	
Physical activity (moderate/vigorous)	Sedentary (0)	443	12.40%		418	12.61%	
	1	479	13.40%		443	13.37%	
	2	1116	31.23%		1042	31.44%	
	3	652	18.24%		603	18.20%	
	Active (4)	884	24.73%		808	24.38%	
Triglyceride (mmol/l)		3574	1.43 (0.69)	0.3–4.5	3314	1.43 (0.69)	0.4–4.5
HDL (mmol/l)		3574	1.60 (0.47)	0.4–4	3314	1.59 (0.47)	0.4–4
LDL (mmol/l)		3574	2.91 (0.98)	0.4–7.6	3314	2.91 (0.98)	0.4–7.6
Limiting longstanding illness	No	2534	70.90%		2329	70.28%	
	Yes	1040	29.10%		985	29.72%	
COVID-19 NHS CORE symptoms	No	3491	97.71%		3231	97.52%	
	Yes	82	2.29%		82	2.48%	
Hospitalization for COVID-19	No	3 561	99.66%		3301	99.64%	
	Yes	12	0.34%		12	0.36%	
CRP (log, ≤20 mg/L)		3 574	0.96 (0.60)	0.01–3.01	-	-	-
CRP (≤20 mg/L)	<3 mg/L	2 732	76.44%		-	-	-
	≥3 mg/L	842	23.56%		-	-	-
Fibrinogen (g/L)		-	-	-	3314	3.23 (0.56)	1.6–6.5
Depressive symptoms (Baseline CES-D)		3 574	1.02 (1.41)	0–7	3314	0.08 (0.26)	0–1
Depressive symptoms (Pandemic CES-D)		3 574	1.56 (1.88)	0–7	3314	0.16 (0.37)	0–1
Depressive symptoms (Baseline CES-D)	<4	3 317	92.81%		3,067	92.55%	
	≥4	257	7.19%		247	7.45%	
Depressive symptoms (Pandemic CES-D)	<4	3 001	83.97%		2773	83.68%	
	≥4	573	16.03%		541	16.32%	

*NHS* National Health Service.

### Associations between inflammation and depressive symptoms during the pandemic

Multivariable analyses are summarized in Table 2. CRP was positively associated with the incidence of depressive symptoms. The crude ORs of 1.69 (95% CI 1.38–2.08, *p* = <0.001) in model 1, adjusted for baseline depression, was reduced to 1.40 (95% CI 1.12–1.73, *p* = 0.003) after full adjustment. This indicates that the odds of elevated depressive symptoms during the COVID-19 crisis were increased by 40% among participants with high CRP concentrations pre-pandemic. Plasma fibrinogen was also associated with depressive symptoms (crude OR = 1.29, 95% CI 1.09–1.52, *p* = 0.003) and remained significant after adjusting for baseline depressive symptoms, age, sex, education, wealth (model 3 OR = 1.23, 95% CI 1.04–1.46, *p* = 0.019) and clinical variables (model 5 OR = 1.22, 95% CI 1.03–1.45, *p* = 0.025). However, associations were attenuated and no longer significant after adjustment of lifestyle factors (model 4 OR = 1.16, 95% CI 0.98–1.38, *p* = 0.085), suggesting that these factors accounted substantially for the relationship between fibrinogen and depressive symptoms. The odds for depressive symptoms for every unit increase in fibrinogen were 1.12 (95% CI 0.94–1.34), which was not significant in the fully adjusted model. The largest reduction in odds was observed in models 3 and 4, with an indication that wealth, physical activity and smoking may partially explain the association between inflammation and depressive symptoms during the pandemic.

**Table 2:** Longitudinal associations between pre-pandemic inflammatory markers and depressive symptoms during the pandemic.

Adjustments	CRP (*n* = 3574)			Fibrinogen (*n* = 3314)		
	OR (SE)	95% CI	*p*	OR (SE)	95% CI	*p*
Model 1: adjusted for baseline depressive symptoms	1.69 (0.18)	1.38–2.08	<0.001	1.29 (0.11)	1.09–1.52	0.003
Model 2: Model 1 + adjustment for age and sex	1.65 (0.17)	1.34–2.03	<0.001	1.26 (0.11)	1.07–1.50	0.007
Model 3: Model 1 + adjustment for education and wealth	1.57 (0.17)	1.27–1.93	<0.001	1.23 (0.11)	1.04–1.46	0.019
Model 4: Model 1 + adjustment for lifestyle variables^a^	1.50 (0.16)	1.22–1.85	<0.001	1.16 (0.10)	0.98–1.38	0.085
Model 5: Model 1 + adjustment for clinical variables^b^	1.59 (0.17)	1.29–1.97	<0.001	1.22 (0.11)	1.03–1.45	0.025
Model 6: adjusted for all covariates^c^	1.40 (0.16)	1.12–1.73	0.003	1.12 (0.10)	0.94–1.34	0.180

*CI* confidence interval, *HDL* high-density lipoprotein, *LDL* low-density lipoprotein, *OR* odds ratio, *p* significance value.

^a^Lifestyle variables = smoking status, alcohol consumption, physical activity.

^b^Clinical variables = triglyceride, HDL, LDL, limiting longstanding illness.

^c^All covariates = depressive symptoms (CES-D ≥ 4), age, sex, education, wealth, smoking status, alcohol consumption, physical activity, triglyceride, HDL, LDL, limiting longstanding illness.

### Sensitivity analyses

The first sensitivity analysis modelled depressive symptoms as continuous scores. Findings did not substantially deviate from the results of the main analyses (Table S1 in the Supplement). The *β* adjusted for baseline depression, age and sex (*β* = 0.23, 95% CI 0.10–0.36, *p* < 0.001) was 0.14 (95% CI 0.01–0.27, *p* = 0.034) in the fully adjusted CRP model. The results for the prospective associations between fibrinogen and depressive symptoms were significant in models 1–5 but no longer robust in the fully adjusted model (*β* = 0.07, 95% CI −0.04 to 0.17, *p* = 0.202). The second sensitivity analysis showed that the associations between CRP and depressive symptoms were mostly unaffected by additional adjustment for coronavirus exposure (Table S2). Estimates of the relationship between fibrinogen and depressive symptoms remained broadly similar after exposure to the coronavirus was taken into account. In the third sensitivity analysis, the magnitude of associations remained unchanged when analyses included very high CRP values. The fourth sensitivity analysis modelled CRP as a continuous measure. The association with depressive symptoms during the pandemic remained significant in the fully adjusted model (OR = 1.18, 95% CI 1.00–1.39, *p* = 0.046; Table S3). The fifth sensitivity analysis introduced BMI as an additional covariate (Table S4). The sample size was reduced both for the CRP and fibrinogen analyses, resulting in reduced power. However, the association between CRP and depressive symptoms (model 5 OR = 1.41, 95% CI 1.12–1.79, *p* = 0.004), and fibrinogen and depressive symptoms (model 5 OR = 1.24, 95% CI 1.03–1.50, *p* = 0.026) remained significant when BMI was added to the models. In sensitivity analysis six, alcohol consumption was modelled across eight categories. The results were mostly unchanged from those of the primary analysis (Table S5). Finally, in the seventh sensitivity analysis, we conditioned on personal exposure to the coronavirus, the financial impact of the pandemic and a difficulty in accessing services during the pandemic. The relationship between CRP and depressed mood (model 6 OR = 1.70, 95% CI 1.38–2.09, *p* < 0.001; Table S6), and fibrinogen and depressed mood (model 6 OR = 1.29, 95% CI 1.09–1.52, *p* = 0.003) was independent of these COVID-19 impact factors.

## Discussion

This study sought to relate the magnitude of change in depressive symptoms during the pandemic in older adults with earlier levels of systemic inflammation, while taking into consideration pre-pandemic levels of depressive symptomatology. The results revealed that pre-pandemic CRP concentrations were positively associated with depressive symptoms in the early months of the COVID-19 pandemic in England, independently of pre-pandemic depression, sociodemographic factors, lifestyle and health-related factors. Pre-pandemic fibrinogen concentration was also related to depressive symptoms during the pandemic, but these associations were explained by covariates, notably lifestyle factors.

Infection with COVID-19 has been linked with subsequent severe psychiatric conditions [3, 37], but even in the general population without COVID-19 infection, increases in psychological distress are substantial [15, 38–40]. Inflammation is relevant to this dynamic for two reasons. First, psychological stress modulates immunity at cellular and molecular levels, as has been established in experimental and observational studies, which can lead to prolonged endocrine and immune dysregulation with deleterious health consequences [41]. Second, systemic inflammation is an important determinant of depressive symptoms, and this association has been established in animal models [42], studies of affective responses to pro-inflammatory medication [43], in addition to population and clinical studies [20–22, 44, 45].

Our results suggest that the background level of systemic inflammation measured before the pandemic is associated with heightened depressive symptoms during the stressful early phase of the pandemic. In the analytic models adjusted for age, sex and baseline symptoms of depression, the odds of depressive symptoms during June/July 2020 increased 69% for high CRP and 29% for each unit increase in fibrinogen. Stress-induced sensitization of the neuroimmune microenvironment [41], neuroendocrine pathways [45] and inflammasomes [46] have been identified as potential mechanisms contributing to these findings. Although CRP and fibrinogen are positively correlated and both are reliable indicators of inflammation, each represent different aspects of inflammation. CRP is known to be a more sensitive neuroimmune biomarker, as patterns of change in plasma fibrinogen concentration are fairly more moderate [47, 48]. This is likely due to fibrinogen being additionally involved in other physiological processes such as haemostasis and angiogenesis [49].

The origin of differences in pre-pandemic inflammatory levels governed the selection of covariates in these analyses. Systemic inflammation is inversely associated with socioeconomic status [26], physical health [33] and lifestyle factors, such as smoking and sedentary behaviour [50]. Smoking and sedentary behaviours are known to have increased in a subset of the population during the COVID-19 pandemic [51, 52], and during this same period, lower socioeconomic status and physical health were shown to predict greater psychological distress [16, 53]. There is additional evidence that smoking [54] and physical inactivity [50] are related to inflammation and depression. Within our sample, lifestyle factors (i.e., smoking, inactivity, and alcohol consumption) had the largest impact on the association between inflammation and depression during the pandemic (Table 2), but even when these and other factors were taken into account, the relationship for CRP remained significant.

Sensitivity analyses confirmed that depressive symptoms as continuous scores did not substantially deviate from the results of the main analyses. In addition, the magnitude of effects remained unchanged when analyses were performed after including individuals with CRP values of 20 mg/L and above. The regression coefficients were only minimally affected by the additional adjustment of subjects who were exposed to the coronavirus. Modelling CRP as a continuous variable provided a similar pattern of results to those of the main findings. We also tested whether personal experiences during the pandemic affected the pattern of results. Exposure to stressors such as financial hardship, restricted access to services and COVID-19 infection among friends and family did not modify the primary results. This is not to imply that these factors do not contribute to psychological distress during the pandemic, but that their influence was independent of the links between inflammation and depression that we identified. These sensitivity analyses ultimately suggest that our conclusions have not been biased by the way that variables have been categorized.

Further research is needed to develop a complete picture of other psychological outcomes experienced during the pandemic due to neuroimmune persistence. In addition, an exploration into other possible biomarkers could offer insight into the extent of biological mechanisms involved. This could confer targets for treatment in inflammation-induced psychiatric conditions and could be especially advantageous in reducing psychological burden during pandemics. Public health systems could become better equipped to manage population distress in the face of potential future widescale virulent outbreaks and, in doing so, the healthcare burden and public spending could be reduced [55].

To the best of our knowledge, this is the first study to prospectively address inflammatory conditions prior to the COVID-19 pandemic in relation to depressed mood during the pandemic, while considering earlier levels of depressive symptomatology. In addition, few studies have explored the role of both CRP and fibrinogen in the experience of depressive symptoms in this context. A significant strength of this study is that we analysed a large, nationally representative sample of older adults. Pre-pandemic measures of inflammation were measured as part of routine data collection before COVID-19 emerged, so prior expectations could not bias results. The response rate for data collection during June/July 2020 (75%) was higher than in most studies of mental health during the pandemic. Despite concerns that the inclusion of participants who had been exposed to the coronavirus may lead to an overestimation of emotional responses, the inclusion of their data did not bias our results.

Nevertheless, our conclusions should be interpreted in light of a number of limitations. We relied on self-reports of depressive symptoms, rather than on clinical diagnosis. Concerns have been raised about the under-reporting of depressive symptoms in older adults [56]. Depressive symptoms were measured early in the pandemic and distress has been shown to fluctuate over time [57]. Moreover, this observational study would have benefited from a more extended follow-up period and the use of time-stratified survival analysis to strengthen inferences. Finally, we cannot be certain of inflammatory levels immediately before the COVID-19 pandemic, as measures were taken 1–3 years earlier. However, other studies have demonstrated that inflammation is relatively stable over several years in the ELSA cohort [58].

In a cohort of UK older adults, we found that those with heightened inflammation before the pandemic were at most risk of developing elevated depressive symptoms in the early months of the COVID-19 crisis. Earlier immune dysfunction may be a key consideration in the development of depressed mood during pandemics where psychosocial stressors are pervasive. The high prevalence of population distress has implications for community mental ill health, public resources, national recovery and preparedness in the face of future virulent outbreaks.

## Supplementary information

Supplementary Tables

## Data Availability

The data are deposited in the UK Data Archive and freely available through the UK Data Service (SN 8688 and 5050) and can be accessed here: https://discover.ukdataservice.ac.uk.

## Code availability

The code of the statistical analyses is available from the corresponding author upon request.
