# Supplementary material for: Systemic inflammation and emotional responses during the COVID-19 pandemic
Source: Transl Psychiatry. 2021 Dec 9;11:626. doi: 10.1038/s41398-021-01753-5 (PMC8656139; doi:10.1038/s41398-021-01753-5)
Supplement: Supplementary file 1 — Supplementary Tables [file 41398_2021_1753_MOESM1_ESM.docx]

**Sensitivity Analyses | Neuro-immune Biomarkers and Depressive Symptoms at Baseline**

Table S1. Longitudinal associations between pre-pandemic inflammatory markers and depressive symptoms during the pandemic modelled as continuous scores

| **Adjustments** | **CRP (*n* = 3 574)** | | | **Fibrinogen (*n* = 3 314)** | | |
| --- | --- | --- | --- | --- | --- | --- |
|  | **Coef. (SE)** | **95% CI** | ***p*** | **Coef. (SE)** | **95% CI** | ***p*** |
| Model 1: *adjusted for baseline*  *depressive symptoms* | 0.25 (0.07) | 0.12-0.38 | <0.001 | 0.14 (0.05) | 0.04-0.24 | 0.005 |
| Model 2: *Model 1 +* *adjustment*  *for age and sex* | 0.23 (0.07) | 0.10-0.36 | <0.001 | 0.13 (0.05) | 0.03-0.23 | 0.013 |
| Model 3: *Model 1 +* *adjustment*  *for education and wealth* | 0.21 (0.07) | 0.08-0.34 | 0.001 | 0.13 (0.05) | 0.03-0.23 | 0.014 |
| Model 4: *Model 1 +* *adjustment for lifestyle* *variables*^a^ | 0.20 (0.07) | 0.07-0.33 | 0.003 | 0.11 (0.05) | 0.00-0.21 | 0.044 |
| Model 5: *Model 1 +* *adjustment for clinical variables*^b^ | 0.21 (0.07) | 0.08-0.34 | 0.001 | 0.11 (0.05) | 0.01-0.22 | 0.028 |
| Model 6: *adjusted for all covariates*^c^ | 0.14 (0.07) | 0.01-0.27 | 0.034 | 0.07 (0.05) | -0.04-0.17 | 0.202 |
| *Note*. Regression coefficient (X) = a one-unit increase in inflammation is associated with an X unit increase in depressive symptoms; SE = standard error; CI = confidence interval; *p* = significance value.  a *Lifestyle variables* = smoking status; alcohol consumption; physical activity.  b *Clinical variables* = triglyceride; high-density lipoprotein (HDL); low-density lipoprotein (LDL); limiting longstanding illness.  c All covariates = depressive symptoms (CES-D); age; sex; education; wealth; smoking status; alcohol consumption; physical activity; triglyceride; high-density lipoprotein (HDL); low-density lipoprotein (LDL); limiting longstanding illness. | | | | | | |

Table S2. Longitudinal associations between pre-pandemic inflammatory markers and depressive symptoms during the pandemic, accounting for participant exposure to the coronavirus

| **Adjustments** | **CRP (*n* = 3 573)** | | | **Fibrinogen (*n* = 3 313)** | | |  |
| --- | --- | --- | --- | --- | --- | --- | --- |
|  | **Coef. (SE)** | **95% CI** | ***p*** | **Coef. (SE)** | **95% CI** | ***p*** |  |
| Model 1: *adjusted for baseline*  *depressive symptoms* | 0.25 (0.07) | 0.12-0.38 | <0.001 | 0.14 (0.05) | 0.04-0.24 | 0.005 |  |
| Model 2: *Model 1 +* *adjustment*  *for age and sex* | 0.23 (0.07) | 0.10-0.36 | <0.001 | 0.13 (0.05) | 0.03-0.23 | 0.014 |  |
| Model 3: *Model 1 +* *adjustment*  *for education and wealth* | 0.21 (0.07) | 0.08-0.34 | 0.001 | 0.13 (0.05) | 0.02-0.23 | 0.016 |  |
| Model 4: *Model 1 +* *adjustment for lifestyle* *variables*^a^ | 0.20 (0.07) | 0.07-0.32 | 0.003 | 0.10 (0.05) | 0.00-0.21 | 0.047 |  |
| Model 5: *Model 1 +* *adjustment for clinical variables*^b^ *and COVID-19 variables*^c^ | 0.21 (0.07) | 0.08-0.34 | 0.001 | 0.11 (0.05) | 0.01-0.22 | 0.029 |  |
| Model 6: *adjusted for all covariates*^d^ | 0.14 (0.07) | 0.01-0.27 | 0.034 | 0.07 (0.05) | -0.04-0.17 | 0.216 |  |
| *Note*. Regression coefficient (X) = one-unit increase in inflammation is associated with an X unit increase in depressive symptoms; *SE* = standard error; CI = confidence interval; *p* = significance value.  a *Lifestyle variables* = smoking status; alcohol consumption; physical activity.  b *Clinical variables* = triglyceride; high-density lipoprotein (HDL); low-density lipoprotein (LDL); limiting longstanding illness.  c *COVID-19 variables* = hospitalized for COVID-19; two of three National Health Service (NHS) core coronavirus symptoms.  d All covariates = depressive symptoms (CES-D); age; sex; education; wealth; smoking status; alcohol consumption; physical activity; triglyceride; high-density lipoprotein (HDL); low-density lipoprotein (LDL); limiting longstanding illness; hospitalized for COVID-19; two of three National Health Service (NHS) core coronavirus symptoms. | | | | | | | |

Table S3. Longitudinal associations between inflammatory markers (continuous logarithmic transformed CRP) and depressive symptoms during the pandemic

| **Adjustments** | **CRP (*n* = 3 574)** | | |
| --- | --- | --- | --- |
|  | **OR (*SE*)** | **95% CI** | ***p*** |
| Model 1: *adjusted for baseline*  *depressive symptoms* | 1.39 (0.11) | 1.19-1.61 | <0.001 |
| Model 2: *Model 1 +* *adjustment*  *for age and sex* | 1.35 (0.10) | 1.16-1.57 | <0.001 |
| Model 3: *Model 1 +* *adjustment*  *for education and wealth* | 1.30 (0.10) | 1.12-1.52 | 0.001 |
| Model 4: *Model 1 +* *adjustment for*  *lifestyle* *variables*^a^ | 1.26 (0.10) | 1.08-1.47 | 0.004 |
| Model 5: *Model 1 +* *adjustment for*  *clinical variables*^b^ | 1.32 (0.11) | 1.13-1.55 | <0.001 |
| Model 6: *adjusted for all covariates*^d^ | 1.18 (0.10) | 1.00-1.39 | 0.046 |
| *Note*. OR = (odds ratio); *SE* = standard error; CI = confidence interval;  *p* = significance value.  a *Lifestyle variables* = smoking status; alcohol consumption; physical activity.  b *Clinical variables* = triglyceride; high-density lipoprotein (HDL); low-density lipoprotein (LDL); limiting longstanding illness.  c All covariates = depressive symptoms (CES-D ≥4); age; sex; education; wealth; smoking status; alcohol consumption; physical activity; triglyceride; high-density lipoprotein (HDL); low-density lipoprotein (LDL); limiting longstanding illness. | | | |

Table S4. Longitudinal associations between pre-pandemic inflammatory markers and depressive symptoms during the pandemic, accounting for BMI.

| **Adjustments** | **CRP (*n* = 3 120)** | | | **Fibrinogen (*n* = 2 880)** | | |  |
| --- | --- | --- | --- | --- | --- | --- | --- |
|  | **OR (*SE*)** | **95% CI** | ***p*** | **OR (*SE*)** | **95% CI** | ***p*** |  |
| Model 1: *adjusted for baseline*  *depressive symptoms* | 1.56 (0.18) | 1.24-1.95 | <0.001 | 1.33 (0.12) | 1.11-1.60 | 0.002 |  |
| Model 2: *Model 1 +* *adjustment*  *for age and sex* | 1.52 (0.18) | 1.22-1.91 | <0.001 | 1.29 (0.12) | 1.08-1.56 | 0.006 |  |
| Model 3: *Model 1 +* *adjustment*  *for education and wealth* | 1.45 (0.17) | 1.16-1.82 | <0.001 | 1.27 (0.12) | 1.06-1.53 | 0.010 |  |
| Model 4: *Model 1 +* *adjustment for lifestyle* *variables*^a^ | 1.38 (0.16) | 1.10-1.74 | 0.006 | 1.21 (0.12) | 1.00-1.46 | 0.046 |  |
| Model 5: *Model 1 +* *adjustment for clinical variables*^b^ *and BMI*^c^ | 1.41 (0.17) | 1.12-1.79 | 0.004 | 1.24 (0.12) | 1.03-1.50 | 0.026 |  |
| Model 6: *adjusted for all covariates*^d^ | 1.26 (0.16) | 0.99-1.61 | 0.056 | 1.14 (0.11) | 0.94-1.39 | 0.189 |  |
| *Note*. OR = (odds ratio); *SE* = standard error; CI = confidence interval; *p* = significance value.  a *Lifestyle variables* = smoking status; alcohol consumption; physical activity.  b *Clinical variables* = triglyceride; high-density lipoprotein (HDL); low-density lipoprotein (LDL); limiting longstanding illness; BMI.  c All covariates = depressive symptoms (CES-D ≥4); age; sex; education; wealth; smoking status; alcohol consumption; physical activity; triglyceride; high-density lipoprotein (HDL); low-density lipoprotein (LDL); limiting longstanding illness; BMI. | | | | | | | |

Table S5. Longitudinal associations between pre-pandemic inflammatory markers and depressive symptoms during the pandemic, conditioned on an 8-point classification of alcohol consumption

| **Adjustments** | **CRP (*n* = 3 574)** | | | **Fibrinogen (*n* = 3 331)** | | |  |
| --- | --- | --- | --- | --- | --- | --- | --- |
|  | **OR (*SE*)** | **95% CI** | ***p*** | **OR (*SE*)** | **95% CI** | ***p*** |  |
| Model 1: *adjusted for baseline*  *depressive symptoms* | 1.69 (0.18) | 1.38-2.08 | <0.001 | 1.29 (0.11) | 1.09-1.52 | 0.003 |  |
| Model 2: *Model 1 +* *adjustment*  *for age and sex* | 1.65 (0.17) | 1.34-2.03 | <0.001 | 1.26 (0.11) | 1.07-1.50 | 0.007 |  |
| Model 3: *Model 1 +* *adjustment*  *for education and wealth* | 1.57 (0.17) | 1.27-1.93 | <0.001 | 1.23 (0.11) | 1.04-1.46 | 0.019 |  |
| Model 4: *Model 1 +* *adjustment for*  *lifestyle* *variables*^a^ | 1.49 (0.16) | 1.21-1.84 | <0.001 | 1.14 (0.10) | 0.96-1.36 | 0.136 |  |
| Model 5: *Model 1 +* *adjustment for*  *clinical variables*^b^ | 1.59 (0.17) | 1.29-1.97 | <0.001 | 1.22 (0.11) | 1.03-1.45 | 0.025 |  |
| Model 6: *adjusted for all covariates*^c^ | 1.40 (0.16) | 1.13-1.75 | 0.002 | 1.12 (0.10) | 0.93-1.34 | 0.224 |  |
| *Note*. OR = (odds ratio); *SE* = standard error; CI = confidence interval; *p* = significance value.  a *Lifestyle variables* = smoking status; alcohol consumption; physical activity.  b *Clinical variables* = triglyceride; high-density lipoprotein (HDL); low-density lipoprotein (LDL); limiting longstanding illness.  c All covariates = depressive symptoms (CES-D ≥4); age; sex; education; wealth; smoking status; alcohol consumption; physical activity; triglyceride; high-density lipoprotein (HDL); low-density lipoprotein (LDL); limiting longstanding illness. | | | | | | | |

Table S6. Longitudinal associations between pre-pandemic inflammatory markers and depressive symptoms during the pandemic, accounting for participant exposure to the coronavirus, financial impact of the pandemic and a difficulty in accessing services during the pandemic

| **Adjustments** | **CRP (*n* = 3 572)** | | | **Fibrinogen (*n* = 3 312)** | | |  |
| --- | --- | --- | --- | --- | --- | --- | --- |
|  | **OR (*SE*)** | **95% CI** | ***p*** | **OR (*SE*)** | **95% CI** | ***p*** |  |
| Model 1: *adjusted for baseline*  *depressive symptoms* | 1.70 (0.18) | 1.38-2.08 | <0.001 | 1.29 (0.11) | 1.09-1.53 | 0.003 |  |
| Model 2: *Model 1 +* *adjustment*  *for age and sex* | 1.65 (0.17) | 1.34-2.03 | <0.001 | 1.27 (0.11) | 1.07-1.50 | 0.007 |  |
| Model 3: *Model 1 +* *adjustment*  *for education and wealth* | 1.57 (0.17) | 1.28-1.94 | <0.001 | 1.23 (0.11) | 1.04-1.46 | 0.016 |  |
| Model 4: *Model 1 +* *adjustment for*  *lifestyle* *variables*^a^ | 1.51 (0.16) | 1.22-1.86 | <0.001 | 1.16 (0.10) | 0.98-1.38 | 0.084 |  |
| Model 5: *Model 1 +* *adjustment for*  *clinical variables*^b^ | 1.60 (0.17) | 1.29-1.97 | <0.001 | 1.23 (0.11) | 1.03-1.46 | 0.020 |  |
| Model 6: *Model 1 + COVID-19 impact variables*^c^ | 1.70 (0.18) | 1.38-2.09 | <0.001 | 1.29 (0.11) | 1.09-1.52 | 0.003 | |
| Model 7: *adjusted for all covariates*^d^ | 1.40 (0.16) | 1.13-1.74 | 0.003 | 1.12 (0.10) | 0.94-1.34 | 0.215 |  |
| *Note*. OR = (odds ratio); *SE* = standard error; CI = confidence interval; *p* = significance value.  a *Lifestyle variables* = smoking status; alcohol consumption; physical activity.  b *Clinical variables* = triglyceride; high-density lipoprotein (HDL); low-density lipoprotein (LDL); limiting longstanding illness.  c *COVID-19 impact variables* = exposure to the coronavirus, the financial impact of the pandemic and a difficulty in accessing services during the pandemic.  d All covariates = depressive symptoms (CES-D ≥4); age; sex; education; wealth; smoking status; alcohol consumption; physical activity; triglyceride; high-density lipoprotein (HDL); low-density lipoprotein (LDL); limiting longstanding illness. | | | | | | | |
